# Time- and Region-Specific Selection of Reference Genes in the Rat Brain in the Lithium–Pilocarpine Model of Acquired Temporal Lobe Epilepsy

**DOI:** 10.3390/biomedicines12051100

**Published:** 2024-05-16

**Authors:** Alexander P. Schwarz, Maria V. Zakharova, Anna A. Kovalenko, Alexandra V. Dyomina, Olga E. Zubareva, Aleksey V. Zaitsev

**Affiliations:** Sechenov Institute of Evolutionary Physiology and Biochemistry of Russian Academy of Sciences, Toreza Prospekt, 44, 194223 Saint Petersburg, Russia; zaharova-masha@yandex.ru (M.V.Z.); kovalenko_0911@mail.ru (A.A.K.); adyomina513@gmail.com (A.V.D.); zubarevaoe@mail.ru (O.E.Z.)

**Keywords:** reference gene expression stability, lithium–pilocarpine model, brain, rat, acquired epilepsy, quantitative reverse transcription polymerase chain reaction

## Abstract

Reverse transcription followed by quantitative polymerase chain reaction (RT-qPCR) is a commonly used tool for gene expression analysis. The selection of stably expressed reference genes is required for accurate normalization. The aim of this study was to identify the optimal reference genes for RT-qPCR normalization in various brain regions of rats at different stages of the lithium–pilocarpine model of acquired epilepsy. We tested the expression stability of nine housekeeping genes commonly used as reference genes in brain research: *Actb*, *Gapdh*, *B2m*, *Rpl13a*, *Sdha*, *Ppia*, *Hprt1*, *Pgk1*, and *Ywhaz*. Based on four standard algorithms (geNorm, NormFinder, BestKeeper, and comparative delta-Ct), we found that after pilocarpine-induced status epilepticus, the stability of the tested reference genes varied significantly between brain regions and depended on time after epileptogenesis induction (3 and 7 days in the latent phase, and 2 months in the chronic phase of the model). *Pgk1* and *Ywhaz* were the most stable, while *Actb*, *Sdha*, and *B2m* demonstrated the lowest stability in the analyzed brain areas. We revealed time- and region-specific changes in the mRNA expression of the housekeeping genes *B2m*, *Actb*, *Sdha*, *Rpl13a*, *Gapdh*, *Hprt1*, and *Sdha.* These changes were more pronounced in the hippocampal region during the latent phase of the model and are thought to be related to epileptogenesis. Thus, RT-qPCR analysis of mRNA expression in acquired epilepsy models requires careful selection of reference genes depending on the brain region and time of analysis. For the time course study of epileptogenesis in the rat lithium–pilocarpine model, we recommend the use of the *Pgk1* and *Ywhaz* genes.

## 1. Introduction

Epilepsy is a common chronic neurological disorder with a high incidence of drug-resistant forms [1,2]. It is the subject of active study in both clinical and experimental settings. The lithium–pilocarpine temporal lobe epilepsy model is one of the most commonly used animal models to study the pathogenesis of acquired epilepsy [3]. It shares many morphofunctional characteristics with human disease and is characterized by a latent phase in which spontaneous recurrent seizures do not occur in animals [3,4,5]. Pathogenic processes can lead to changes in the expression levels of various genes. Reverse transcription followed by quantitative polymerase chain reaction (RT-qPCR) is the gold standard and a powerful tool for low-throughput mRNA expression analysis. However, obtaining reliable results requires high-quality data [6]. An important issue in RT-qPCR mRNA expression analysis is the careful selection of appropriate reference genes for data normalization [7]. An inadequate normalization strategy may result in inaccurate calculations and consequently erroneous results, and may even change the direction of the observed expression changes [8,9,10].

We recently optimized a set of multiplex qPCR assays to quickly analyze the expression of nine commonly used rat reference genes. The chosen reference genes were *Actb*, *Gapdh*, *B2m*, *Rpl13a*, *Ppia*, *Sdha*, *Hprt1*, *Pgk1*, and *Ywhaz* [11]. These genes were selected because they have been shown previously to be stably expressed in brain tissue in different studies, depending on the experimental setup and region [7]. We also tried to choose reference genes connected with different intracellular functions to avoid possible coregulatory bias [11]. The expression of commonly used reference genes may be altered under certain conditions. For example, in a rat mild ketogenic diet model, *Sdha* was downregulated, while *Gapdh* mRNA expression was upregulated in the medial prefrontal cortex [12]. In a rat valproic acid model of autism spectrum disorder, *Actb* mRNA expression was increased, and *Gapdh* was decreased in the hippocampus [9]. Epileptogenesis affects various aspects of brain tissue homeostasis [13,14,15], potentially by altering the expression of housekeeping genes commonly used as a reference. The present study aimed to validate optimal reference genes in the rat brain during the latent and chronic phases of the lithium–pilocarpine temporal lobe epilepsy model and to determine whether the expression of commonly used reference genes is altered in the rat brain in this model.

## 2. Materials and Methods

### 2.1. Animals and the Lithium–Pilocarpine Model of Temporal Lobe Epilepsy

Experiments were conducted on 7-week-old male Wistar rats (*n* = 69 rats, 37 in the control group and 32 in the pilocarpine-treated group) following the Animal Care and Use Committee rules of the Sechenov Institute of Evolutionary Physiology and Biochemistry of RAS and the EU Directive 2010/63/EU for animal experimentation. The animals were used in our previous study and a detailed description of the lithium–pilocarpine model of temporal lobe epilepsy can be found there [16]. Briefly, rats premedicated with LiCl (1 day before, 127 mg/kg, i.p., Sigma-Aldrich, St. Louis, MO, USA) and (−)-scopolamine methyl bromide (1 h before, 1 mg/kg, i.p., Sigma-Aldrich) were intermittently injected with pilocarpine (10 mg/kg with 30 min intervals, i.p. up to 40 mg/kg, Sigma-Aldrich) to reach grade 4 on the Racine scale [17]. The severity of seizures was scored as described before according the modified Racine scale: facial automatism (1), head nodding (2), forelimb myoclonus (3), rearing (4), rearing and falling (5), wild running (6), and generalized clonic–tonic convulsions (7) [17,18]. The seizures were stopped with diazepam (10 mg/kg, i.p., Sigma-Aldrich) after 75 min.

Two months following the induction of seizures, a video recording of the rats’ free behavior was made to evaluate the presence of spontaneous recurrent seizures. Each rat was placed into a transparent cage with water and food for three consecutive days, and videotaped every day for a total of 40 h. Only rats with at least one episode of motor seizures were included in the experimental group (*n* = 10). A total of seven rats exhibited one episode of spontaneous seizures, one animal exhibited two episodes, one animal exhibited three episodes, and one animal exhibited six episodes of spontaneous seizures during the observation period. The duration of a single seizure episode was 37.4 ± 3.2 s (minimum 26 s, maximum 85 s, *n* = 18). The recorded seizures were graded from 4 to 7 on the Racine scale.

### 2.2. Reverse Transcription Followed by Quantitative PCR (RT-qPCR)

The procedures for brain sample preparation, cDNA synthesis, and qPCR were described in detail previously [16]. Brain samples were collected from animals 3 and 7 days and 2 months after seizures. The following brain structures were selected for the study: the dorsal and ventral regions of the hippocampus, temporal cortex, medial prefrontal cortex, amygdala, and striatum. The brain areas were dissected with a freezing microtome, and RNA was subsequently isolated via acid guanidinium thiocyanate-phenol-chloroform extraction [19] using the ExtractRNA reagent (Evrogen, Moscow, Russia) according to the manufacturer’s instructions. To avoid possible genomic DNA contamination, RNA samples were incubated with 1 unit of RQ1 DNAse (Promega, Madison, WI, USA) for 15 min at 37 °C, followed by lithium chloride precipitation and ethanol washing. The purification quality and yield of RNA were assessed spectrophotometrically based on the 260 nm absorbance and 260/280 absorbance ratio, respectively, using a NanoDrop Lite Spectrophotometer (Thermo Fisher Scientific, Waltham, MA, USA). Only samples with 260/280 ratios in the 1.8–2.1 range were used for cDNA synthesis. For the analysis of reference gene expression in the temporal cortex, dorsal cortex, and ventral hippocampus, we used cDNA obtained in previous experiments.

Total RNA (1 μg, medial prefrontal cortex, amygdala, and striatum), oligo-dT (0.5 µg per 1 µg of RNA), 9-mer random (0.25 µg per 1 µg of RNA) primers (DNA-Synthesis, Moscow, Russia), and M-MLV reverse transcriptase (100 units per 1 µg of RNA; Evrogen) were used for cDNA synthesis. A total of 8 µL of RNA solution was mixed with primers and incubated for 10 min at 70 °C. After that, the microtubes were rapidly cooled to 4 °C for primer annealing. Subsequently, they were mixed with revertase-containing solution and incubated for 1 h at 42 °C followed by 10 min at 65 °C.

All the samples were diluted 10-fold before the PCR step. qPCR was performed in a total volume of 10 µL with 0.8 µL of cDNA, 0.75 units of TaqM polymerase (Alkor Bio, St. Petersburg, Russia), 3.5 mM Mg^2+^, specific forward and reverse primers, and hydrolysis (TaqMan) probes (see Table 1). qPCRs were multiplexed (*Hprt1* + *Pgk1* + *Ywhaz*, *Rpl13a* + *Ppia* + *Sdha*, and *Actb* + *Gapdh* + *B2m)* as previously described [11]. PCR for the S100 calcium-binding protein B gene (*S100b*, an astroglial marker) was also performed. The following sequences were used for *S100b* (5′→3′): forward primer, AAGTCCACACCCAGTCCTCT; reverse primer, AGGCTCCTGGTCACCTTTTG; and probe, HEX-ACACCGAAGCCAGAGAGGACTCCGG-BHQ2. Nucleotides were synthesized by DNA-Synthesis.

PCRs were run in tetraplicates in a C1000 Touch thermal cycler combined with a CFX384 Touch Real-Time PCR Detection System (Bio-Rad, Hercules, CA, USA) simultaneously with no template and no reverse transcription control samples. PCR data were analyzed by CFX Manager 3.1 software (Bio-Rad). The number of quantification cycles (Cqs) was obtained via regression. The samples with Cq standard deviations greater than 0.35 were excluded from further analysis. The raw mean Cqs were imported into the web interface of the RefFinder^®^ online tool (https://blooge.cn/RefFinder/ (accessed on 20 March 2024)). RefFinder^®^ [20] generates its stability ranking based on the geometric mean of the stability ranks obtained by four widespread algorithms: NormFinder [21], comparative deltaCt [22], GeNorm [23], and BestKeeper [24]. The 2^−∆∆Ct^ method [25] was used to calculate the relative *S100b* gene expression.

### 2.3. Statistical Analysis

Statistical processing of the data was carried out in GraphPad Prism 8.0.1 (GraphPad Software, San Diego, CA, USA) and IBM SPSS Statistics 23 (IBM, Armonk, NY, USA). The normality of the data distribution was tested using the Shapiro–Wilk test. Levene’s test was used to assess the equality of variances. Mean comparisons were conducted using two-way ANOVA with Sidak’s post hoc test. Differences were considered significant at *p* ≤ 0.05

## 3. Results

We evaluated the expression stability of nine housekeeping genes in the brains of control and pilocarpine-treated rats. Analysis was carried out in six brain areas at the latent (3 and 7 days after pilocarpine-induced seizures) and chronic (2 months) phases of the model.

**Figure 1:**
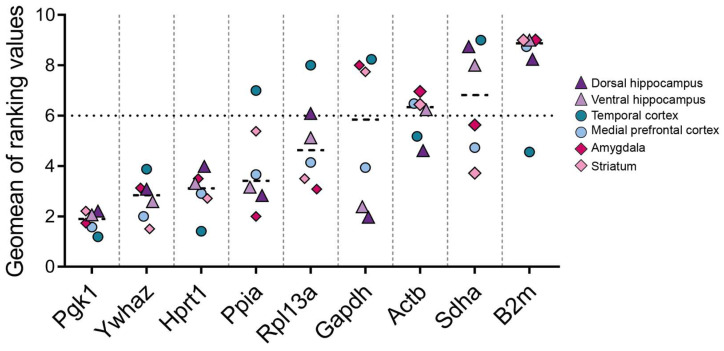
Summary graph of the obtained stability values. Ranking values obtained for nine reference genes in the rat dorsal and ventral hippocampus, temporal and medial prefrontal cortex, striatum, and amygdala in the lithium–pilocarpine temporal lobe epilepsy model. The calculation was performed for overall samples (control and experimental, three time points) within one brain region using the RefFinder^®^ online tool (https://blooge.cn/RefFinder/ (accessed on 20 March 2024)). The dashed line shows the value above which the least stably expressed genes are located.

The first step was to determine the overall stability of the expression of each reference gene in the samples (control and experimental, all time points analyzed). According to the RefFinder^®^ comprehensive rankings based on the stability indices obtained by four algorithms, *Pgk1*, *Ywhaz*, and *Hprt1* were the most stable genes in our model. In contrast, the highest geomean rankings were observed for the genes *B2m*, *Sdha*, and *Actb* (Figure 1).

**Figure 2:**
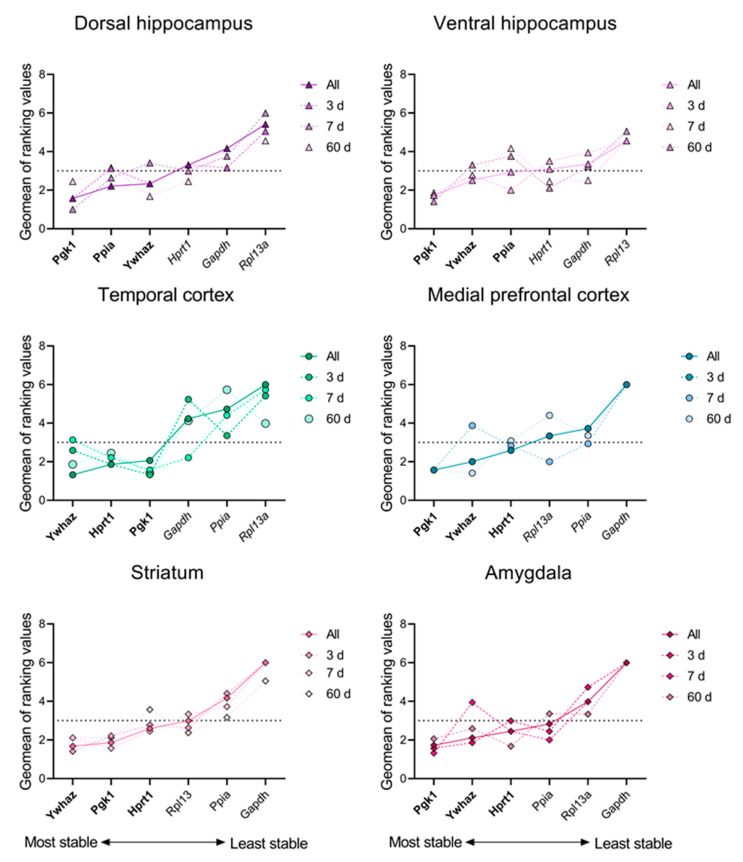
Stability values for various brain regions. The stability ranking of reference genes was assessed in samples from the rat dorsal and ventral hippocampus, temporal and medial prefrontal cortex, striatum, and amygdala in the lithium–pilocarpine model of temporal lobe epilepsy. The stability of mRNA production in all samples from the control and experimental groups was calculated using the RefFinder^®^ online tool (https://blooge.cn/RefFinder/ (accessed on 20 March 2024)) for all time points and separately at 3, 7, and 60 days after pilocarpine-induced seizures.

In the next step of the study, we independently analyzed the reference gene stability rankings for each brain region at 3 days, 7 days, and 2 months after epilepsy induction (Figure 2). To avoid any possible bias of genes with lower stability in the whole set, we performed calculations without *Actb*, *Sdha*, and *B2m*. The stability rankings of six commonly used reference genes analyzed in experimental rats varied across brain regions depending on the time after the induction of epileptogenesis (Figure 2). *Pgk1* and *Ywhaz* were the most stable reference genes in all analyzed brain areas, followed by *Hprt1* in four out of six areas, and *Ppia* was more stable than *Hprt1* in hippocampal tissue (Figure 2; Appendix A Table A1, Table A2, Table A3, Table A4, Table A5 and Table A6).

**Figure 3:**
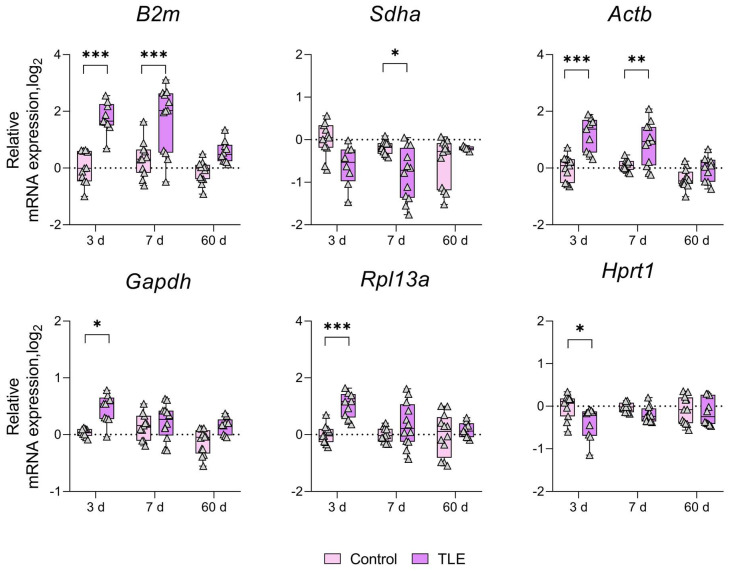
Changes in the expression of commonly used reference genes in the ventral hippocampal area. Analysis was performed 3, 7, and 60 days after pilocarpine-induced status epilepticus. The data were normalized to the expression levels of the three most stable genes (*Pgk1*, *Ywhaz*, and *Ppia*). *, **, *** *p* < 0.05, *p* < 0.01 or *p* < 0.001, respectively (two-way ANOVA followed by Sidak post hoc test); Control—control group, TLE—experimental group. All the data are presented as individual values (triangles) with the minimum, maximum, sample median, and first and third quartiles.

Based on these findings, we investigated whether the mRNA expression of the aforementioned genes changes in the rat brain during pilocarpine-induced epileptogenesis (Table 1). Table 1 presents a summary of the notable alterations (up- or downregulation) in the expression levels of the analyzed genes between the experimental and control groups. Figure 1 presents a graphical representation of the ranking of gene expression stability. Low stability is indicative of an increase in the variability derived from the measured gene expression. In most cases, the mRNA expression of reference genes was up- or downregulated in a region- and time-dependent manner (Table 1). The most prominent changes were observed in the ventral hippocampal area, where all analyzed genes exhibited altered expression levels (Figure 3). During the latent phase of the model (3 and/or 7 days after pilocarpine-induced seizures), the *B2m*, *Actb*, *Gapdh*, and *Rpl13a* mRNAs were upregulated, while the *Sdha* and *Hprt1* mRNAs were downregulated in this area. A long-lasting effect was observed only for *B2m* expression within the amygdala region, where the *B2m* mRNA level increased in the latent and chronic phases of the model (Table 1, Figure A5).

**Figure 4:**
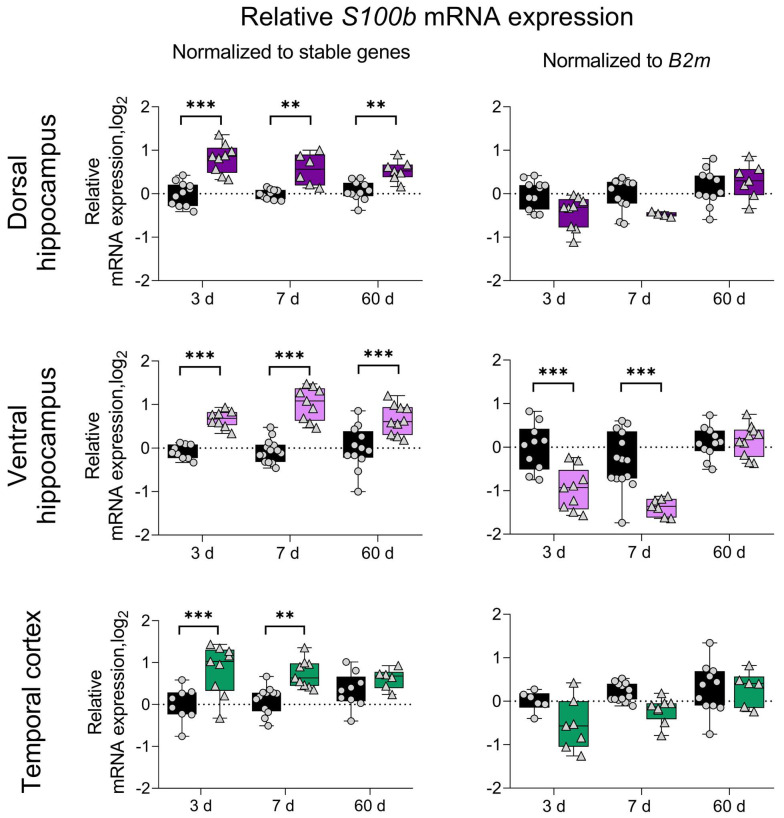
Effects of inappropriate normalization strategies on RT-qPCR results. The use of optimal reference genes revealed that *S100b* mRNA is upregulated in the temporal cortex, ventral and dorsal hippocampus in a rat model of lithium–pilocarpine temporal lobe epilepsy. The use of *B2m* as a reference gene yielded significantly different results. **, *** *p* < 0.01 or *p* < 0.001, respectively (two-way ANOVA followed by Sidak post hoc test). All the data are presented as individual values (circles—control animals, triangles—pilocarpine-treated rats) with the minimum, maximum, sample median, and first and third quartiles.

To illustrate the possible differences in the interpretation of the RT-qPCR data depending on the chosen reference gene, we conducted an expression analysis of the mRNA expression of the astroglial cell marker *S100b* in the dorsal and ventral hippocampus and temporal cortex. The data were normalized to the geometric average of the three most stable reference genes or to the *B2m* mRNA expression. In the present model, *B2m* was identified as the least stable reference gene that was upregulated during the latent phase (Figure 1 and Figure 3, Table 1). The use of optimal reference genes revealed that *S100b* mRNA is upregulated in both the dorsal and ventral hippocampus during the latent and chronic phases of the model. In the temporal cortex, it is only upregulated during the latent phase. Notably, the use of *B2m* as a reference gene yielded significantly different results. Specifically, no differences were detected in the dorsal hippocampus or temporal cortex between the treatment and control groups. At the same time, opposite effects were observed in the ventral hippocampus during the latent phase, as the use of *B2m* showed a decrease in *S100b* mRNA expression (Figure 4).

## 4. Discussion

In the present study, we analyzed the expression of nine of the most frequently used reference genes in different rat brain regions in the lithium–pilocarpine temporal lobe epilepsy model. We observed time- and region-specific changes in the expression of seven genes (*Actb*, *Gapdh*, *B2m*, *Rpl13a*, *Sdha*, *Ppia*, and *Hprt1*) during the latent phase of the model. It is important to avoid using any of these genes as a reference to prevent bias in the interpretation of RT-qPCR data. *Pgk1* and *Ywhaz* were found to be the most stable genes, so we recommend the use of these genes for time course gene expression studies analyzing changes from the latent period to the chronic period in a lithium-pilocarpine model. However, it is important to note the limitations of our study. In particular, the study was conducted only in male rats, and further investigation is needed to determine whether there are sex-specific peculiarities in brain reference gene expression per se, and especially in the epilepsy model.

Changes in housekeeping gene expression in the brain may be related to their potential role in epileptogenesis or may be the result of perturbations in the cell type composition of brain tissue. Epileptogenesis is associated with neuronal loss, astrogliosis, and microglial activation [3]. *B2m* encodes β-2-microglobulin, a component of the main histocompatibility complex that is enriched in cells of the macrophage lineage, including microglia. Therefore, the increase in *B2m* mRNA during the latent phase in the lithium–pilocarpine temporal lobe epilepsy model may be due not only to changes in transcription levels but also to an increase in the number and/or activation of microglia. No changes were detected in *B2m* expression in the striatum (Table 1, Figure A4), which is believed to have an inhibitory effect and not generate epileptiform activity in temporal lobe epilepsy [26]. The B2M protein can act as an endogenous N-methyl-D-aspartate (NMDA) receptor antagonist, which affects synaptic plasticity [27]. Thus, we speculate that *B2m* overexpression could be one of the mechanisms leading to epilepsy development. However, this hypothesis is beyond the scope of this paper and should be tested in independent studies.

In the lithium–pilocarpine epilepsy model, the commonly used reference genes *Actb* and *Gapdh* [7] were found to be unstable in the rat brain. Additionally, *Rpl13a* mRNA was upregulated during the latent phase of the model, which is consistent with human proteomic data showing a significant upregulation of proteins involved in protein synthesis, including RPL13, in epileptic brain tissue [14]. We may hypothesize that changes in *Rpl13a* expression are specific to epileptogenesis because it is highly stable within the rat brain in the pentylenetetrazole-induced seizure model, i.e., single generalized seizures without induction of epileptogenesis [28], as well as in the hippocampus and temporal cortex of healthy dogs and animals with naturally occurring epilepsy [29]. 

In contrast, the genes *Pgk1* and *Ywhaz* were found to be stably expressed in all brain regions analyzed. This indicates that their molecular pathways are not significantly affected by epileptogenesis-related pathophysiological events at the transcriptional level. *Pgk1* encodes the glycolytic enzyme phosphoglycerate kinase 1, while *Ywhaz* encodes tyrosine 3-monooxygenase/tryptophan 5-monooxygenase activation protein zeta (YWHAZ), which plays a central role in many signal transduction pathways [30]. In the pentylenetetrazole model of acute seizures, *Pgk1* was identified as one of the most stable reference genes in the rat brain [28], which is consistent with the findings of the present study. However, in contrast to the present study, the expression of *B2m*, *Sdha*, and *Rpl13a* was found to be stable in most regions of rat brain in the pentylenetetrazole model [28]. These results suggest that changes in the expression of these genes are specific to epileptogenesis rather than acute seizures themselves.

In the lithium–pilocarpine temporal lobe epilepsy model, reference gene expression stability depends on the time point analyzed after epileptogenesis induction. In most cases, there are different optimal choices of three reference genes for 3 or 7 days in the latent stage and for two months in the chronic stage of the model (Figure 2). For example, *Gapdh* is one of the most stable reference genes within the ventral hippocampus and temporal cortex for mRNA expression analysis at 7 days but not at 3 days or 2 months after the induction of epileptogenesis, while *Hprt1* is stably expressed in both hippocampal regions during the chronic but not the latent period of the model (Figure 2). Notably, the reference gene stability changes not only between the latent and chronic periods of the model but also during the latent phase. This could be related to the different pathophysiological processes that occur during epileptogenesis. The reference gene stability rankings as well as the extent of their expression changes in our study were region-specific (Figure 2). These changes were more pronounced in the hippocampal regions, temporal cortex, and amygdala during the latent phase of the model. This may be related to the pathogenic changes associated with epilepsy development. The hippocampus and amygdala are often found to be seizure-promoting brain areas [3,31,32].

RT-qPCR data may be misinterpreted if the mRNA expression level of the reference gene not only shows high variability in the experimental setup but also shows significant changes due to the experimental conditions. We demonstrated that an inappropriate reference gene could even reverse the direction of the observed changes by measuring the mRNA expression of the astroglial marker *S100b* in the hippocampus and temporal cortex against *B2m* or the three most stable reference genes (Figure 4).

Thus, *Pgk1* and *Ywhaz* could be used for time course gene expression analysis of brain changes from the latent period to the chronic period in a rat lithium–pilocarpine temporal lobe epilepsy model. We revealed time- and region-specific changes in the mRNA expression of the housekeeping genes *B2m*, *Actb*, *Sdha*, *Rpl13a*, *Gapdh*, *Hprt1*, and *Sdha*. Our novel findings on changes in the time course of housekeeping gene expression also provide new perspectives for investigating the role of these genes in epileptogenesis.

## Figures and Tables

**Table 1:** Summary of housekeeping gene expression changes in the rat lithium–pilocarpine temporal lobe epilepsy model, as revealed by normalization to three brain region-specific optimal reference genes.

	Gene	*B2m*	*Sdha*	*Actb*	*Gapdh*	*Rpl13a*	*Ppia*	*Hprt1*
Brain Region								
Temporal Cortex		↑ 3 d		↑ 3 d		↑ 3 d		*
		↑ 7 d						
Ventral Hippocampus		↑ 3 d		↑ 3 d	↑ 3 d	↑ 3 d	*	↓ 3 d
		↑ 7 d	↓ 7 d	↑ 7 d				
Dorsal Hippocampus		↑ 3 d		↑ 3 d	↑ 3 d	↑ 3 d	*	↓ 3 d
		↑ 7 d		↑ 7 d	↑ 7 d	↑ 7 d		
Striatum				↑ 3 d	↑ 3 d			*
Amygdala		↑ 3 d		↑ 3 d		↑ 3 d		*
		↑ 7 d		↑ 7 d	↑ 7 d			
		↑ 60 d						
Medial Prefrontal Cortex		↑ 3 d		↑ 3 d		↑ 3 d		*

**Figure A1:**
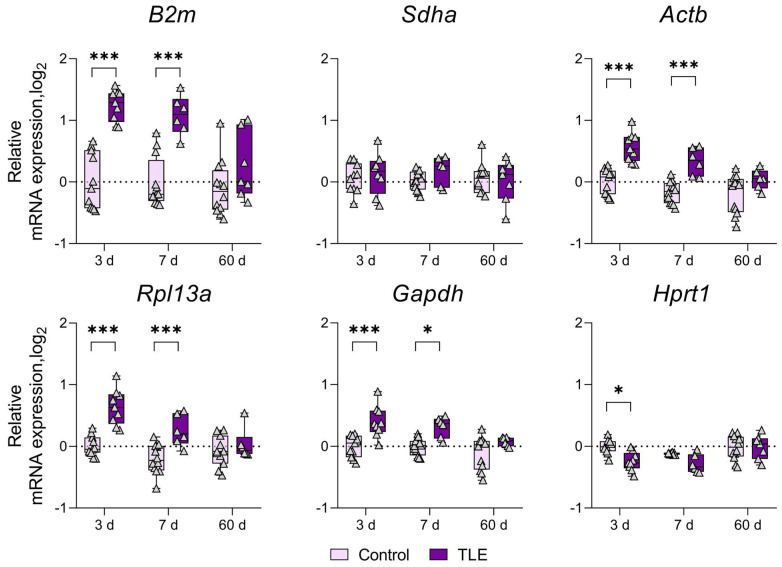
Changes in the expression of commonly used reference genes in the dorsal hippocampal area. Analysis was performed 3, 7, and 60 days after pilocarpine-induced status epilepticus. The data were normalized to the expression levels of the three most stable genes (*Pgk1*, *Ppia*, and *Ywhaz*) in the rat lithium–pilocarpine model of temporal lobe epilepsy. *, *** *p* < 0.05 or *p* < 0.001, respectively (two-way ANOVA followed by Sidak post hoc test); Control—control group, TLE—experimental group. All the data are presented as individual values (triangles) with the minimum, maximum, sample median, and first and third quartiles.

**Figure A2:**
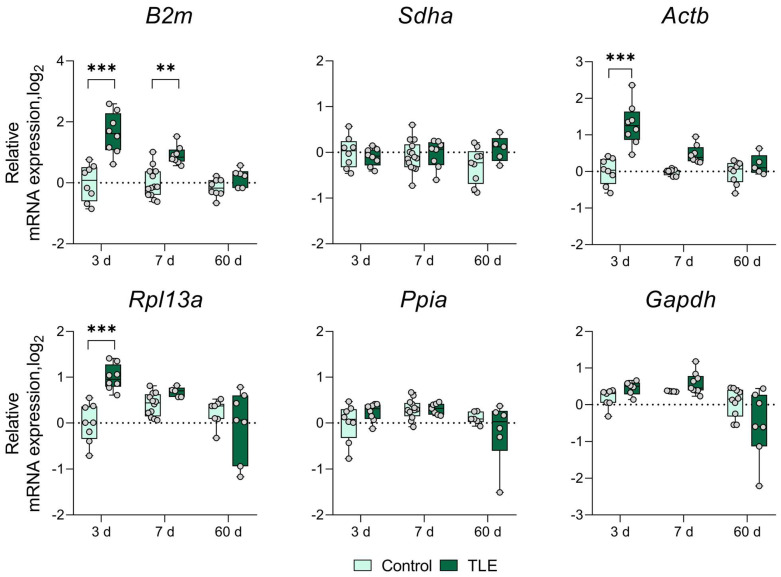
Changes in the expression of commonly used reference genes in the temporal cortex area. Analysis was performed 3, 7, and 60 days after pilocarpine-induced status epilepticus. The data were normalized to the expression levels of the three most stable genes (*Ywhaz*, *Hprt1*, and *Pgk1*) in the rat lithium–pilocarpine model of temporal lobe epilepsy. **, *** *p* < 0.01 or *p* < 0.001, respectively (two-way ANOVA followed by Sidak post hoc test); Control—control group, TLE—experimental group. All the data are presented as individual values (circles) with the minimum, maximum, sample median, and first and third quartiles.

**Figure A3:**
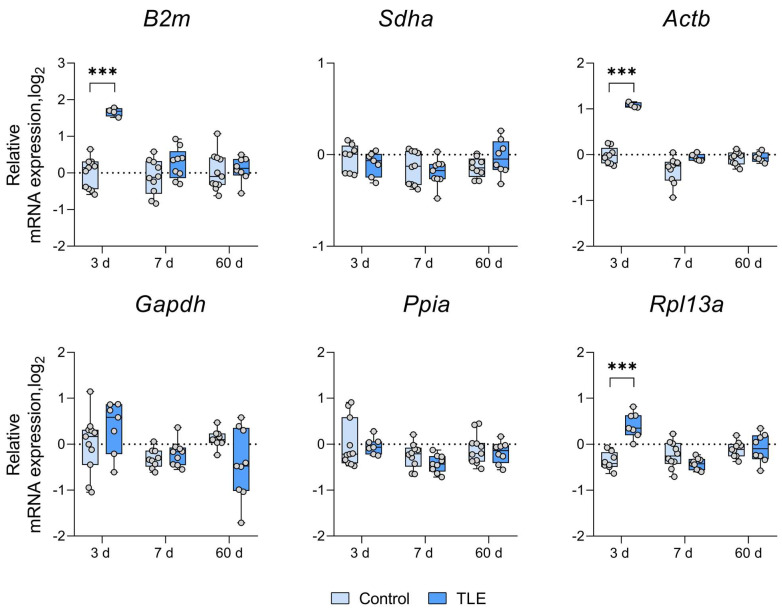
Changes in the expression of commonly used reference genes in the medial prefrontal cortex area. Analysis was performed 3, 7, and 60 days after pilocarpine-induced status epilepticus. The data were normalized to the expression levels of the three most stable genes (*Pgk1*, *Ywhaz*, and *Hprt1*) in the rat lithium–pilocarpine model of temporal lobe epilepsy. *** *p* < 0.001 (two-way ANOVA followed by Sidak post hoc test); Control—control group, TLE—experimental group. All the data are presented as individual values (circles) with the minimum, maximum, sample median, and first and third quartiles.

**Figure A4:**
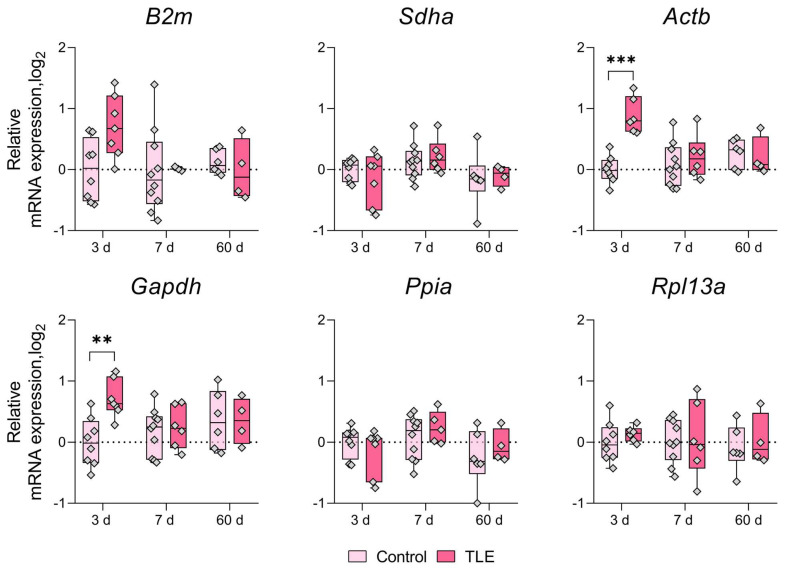
Changes in the expression of commonly used reference genes in the striatum area. Analysis was performed 3, 7, and 60 days after pilocarpine-induced status epilepticus. The data were normalized to the expression levels of the three most stable genes (*Pgk1*, *Ywhaz*, and *Hprt1*) in the rat lithium–pilocarpine model of temporal lobe epilepsy. **, *** *p* < 0.01 or *p* < 0.001, respectively (two-way ANOVA followed by Sidak post hoc test); Control—control group, TLE—experimental group. All the data are presented as individual values (rhombuses) with the minimum, maximum, sample median, and first and third quartiles.

**Figure A5:**
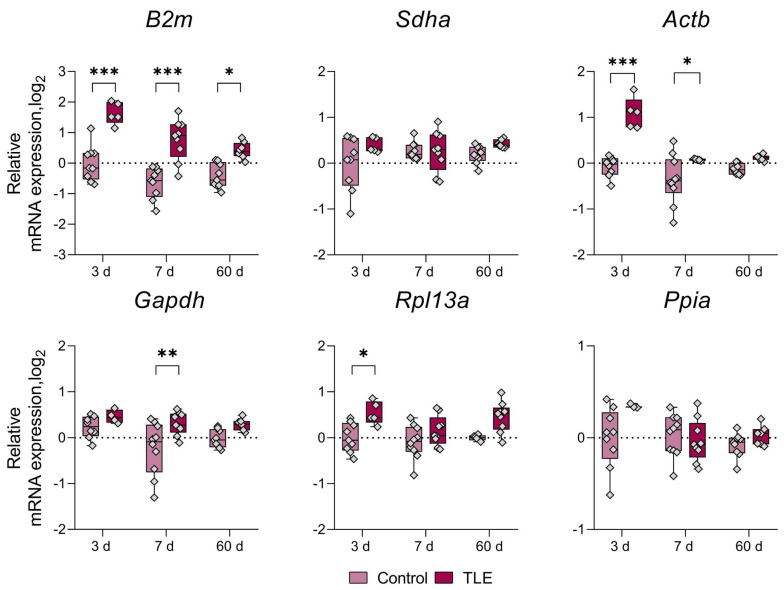
Changes in the expression of commonly used reference genes in the amygdala area. Analysis was performed 3, 7, and 60 days after pilocarpine-induced status epilepticus. The data were normalized to the expression levels of the three most stable genes (*Pgk1*, *Ywhaz*, and *Hprt1*) in the rat lithium–pilocarpine model of temporal lobe epilepsy. *, **, *** *p* < 0.05, *p* < 0.01 or *p* < 0.001, respectively (two-way ANOVA followed by Sidak post hoc test); Control—control group, TLE—experimental group. All the data are presented as individual values (rhombuses) with the minimum, maximum, sample median, and first and third quartiles.

↑ ↓ upregulation or downregulation, respectively, of corresponding gene mRNA observed 3 days (3 d), 7 days (7 d), or 2 months (60 d) after lithium-pilocarpine induced seizures; detailed data are presented in Appendix A (Figure A1, Figure A2, Figure A3, Figure A4 and Figure A5). * marked genes were among the three most stable genes in certain regions; detailed data are presented in Appendix A (Figure A1, Figure A2, Figure A3, Figure A4 and Figure A5).

## Data Availability

The raw data supporting the conclusions of this article will be made available by the authors on request.

## Appendix A

**Table A1:** Reference gene stability values within the amygdala of rats in the latent and chronic phases of the lithium–pilocarpine temporal lobe epilepsy model obtained by four algorithms with the RefFinder online tool.

Amygdala										
	Delta CT		BestKeeper		NormFinder		GeNorm		Comprehensive Ranking	
Rank	Gene	Average of STDV	Gene	Std Dev	Gene	Stability Value	Gene	Stability Value	Gene	Geomean of Ranking Values
1	*Pgk1*	0.32	*Ppia*	0.19	*Pgk1*	0.089	*Hprt1* | *Ywhaz*	0.215	*Pgk1*	1.73
2	*Ywhaz*	0.35	*Rpl13a*	0.21	*Ywhaz*	0.203			*Ywhaz*	2.11
3	*Hprt1*	0.35	*Pgk1*	0.24	*Hprt1*	0.216	*Pgk1*	0.228	*Hprt1*	2.45
4	*Ppia*	0.36	*Hprt1*	0.31	*Ppia*	0.228	*Ppia*	0.268	*Ppia*	2.83
5	*Rpl13a*	0.42	*Ywhaz*	0.33	*Rpl13a*	0.328	*Rpl13a*	0.31	*Rpl13a*	3.98
6	*Gapdh*	0.56	*Gapdh*	0.44	*Gapdh*	0.512	*Gapdh*	0.392	*Gapdh*	6

**Table A2:** Reference gene stability values within the medial prefrontal cortex of rats in the latent and chronic phases of the lithium–pilocarpine temporal lobe epilepsy model obtained by four algorithms with the RefFinder online tool.

Medial Prefrontal Cortex										
	Delta CT		BestKeeper		NormFinder		GeNorm		Comprehensive Ranking	
Rank	Gene	Average of STDV	Gene	Std Dev	Gene	Stability Value	Gene	Stability Value	Gene	Geomean of Ranking Values
1	*Pgk1*	0.36	*Rpl13a*	0.23	*Pgk1*	0.122	*Hprt1 | Ywhaz*	0.175	*Pgk1*	1.57
2	*Ywhaz*	0.37	*Pgk1*	0.24	*Ywhaz*	0.165			*Ywhaz*	2
3	*Hprt1*	0.39	*Ppia*	0.25	*Hprt1*	0.237	*Pgk1*	0.193	*Hprt1*	2.59
4	*Ppia*	0.43	*Ywhaz*	0.32	*Ppia*	0.289	*Ppia*	0.299	*Rpl13a*	3.34
5	*Rpl13a*	0.49	*Hprt1*	0.37	*Rpl13a*	0.411	*Rpl13a*	0.351	*Ppia*	3.72
6	*Gapdh*	0.63	*Gapdh*	0.52	*Gapdh*	0.58	*Gapdh*	0.445	*Gapdh*	6

**Table A3:** Reference gene stability values within the striatum of rats in the latent and chronic phases of the lithium–pilocarpine temporal lobe epilepsy model obtained by four algorithms with the RefFinder online tool.

Striatum										
	Delta CT		BestKeeper		NormFinder		GeNorm		Comprehensive Ranking	
Rank	Gene	Average of STDV	Gene	Std Dev	Gene	Stability Value	Gene	Stability Value	Gene	Geomean of Ranking Values
1	*Ywhaz*	0.33	*Rpl13a*	0.27	*Pgk1*	0.154	*Hprt1 | Ywhaz*	0.164	*Ywhaz*	1.68
2	*Pgk1*	0.34	*Pgk1*	0.29	*Ywhaz*	0.164			*Pgk1*	1.86
3	*Hprt1*	0.35	*Ppia*	0.36	*Hprt1*	0.231	*Pgk1*	0.232	*Hprt1*	2.59
4	*Rpl13a*	0.39	*Ywhaz*	0.4	*Rpl13a*	0.289	*Ppia*	0.312	*Rpl13*	2.99
5	*Ppia*	0.4	*Hprt1*	0.44	*Ppia*	0.315	*Rpl13a*	0.327	*Ppia*	4.16
6	*Gapdh*	0.5	*Gapdh*	0.48	*Gapdh*	0.446	*Gapdh*	0.385	*Gapdh*	6

**Table A4:** Reference gene stability values within the temporal cortex of rats in the latent and chronic phases of the lithium–pilocarpine temporal lobe epilepsy model obtained by four algorithms with the RefFinder online tool.

Temporal Cortex										
	Delta CT		BestKeeper		NormFinder		GeNorm		Comprehensive Ranking	
Rank	Gene	Average of STDV	Gene	Std Dev	Gene	Stability Value	Gene	Stability Value	Gene	Geomean of Ranking Values
1	*Ywhaz*	1.36	*Pgk1*	0.45	*Ywhaz*	0.661	*Hprt1 | Ywhaz*	0.239	*Ywhaz*	1.32
2	*Pgk1*	1.39	*Hprt1*	0.5	*Hprt1*	0.74			*Hprt1*	1.86
3	*Hprt1*	1.41	*Ywhaz*	0.53	*Pgk1*	0.776	*Pgk1*	0.318	*Pgk1*	2.06
4	*Gapdh*	1.73	*Ppia*	0.67	*Gapdh*	1.284	*Gapdh*	0.636	*Gapdh*	4.23
5	*Ppia*	2.29	*Gapdh*	0.72	*Ppia*	1.783	*Ppia*	1.332	*Ppia*	4.73
6	*Rpl13a*	2.86	*Rpl13a*	1	*Rpl13a*	2.674	*Rpl13a*	1.841	*Rpl13a*	6

**Table A5:** Reference gene stability values within the dorsal hippocampus of rats in the latent and chronic phases of the lithium–pilocarpine temporal lobe epilepsy model obtained by four algorithms with the RefFinder online tool.

Dorsal Hippocampus										
	Delta CT		BestKeeper		NormFinder		GeNorm		Comprehensive Ranking	
Rank	Gene	Average of STDV	Gene	Std Dev	Gene	Stability Value	Gene	Stability Value	Gene	Geomean of Ranking Values
1	*Pgk1*	0.26	*Ppia*	0.2	*Pgk1*	0.115	*Hprt1 | Ywhaz*	0.134	*Pgk1*	1.57
2	*Ywhaz*	0.27	*Pgk1*	0.2	*Ppia*	0.153			*Ppia*	2.21
3	*Ppia*	0.28	*Gapdh*	0.21	*Ywhaz*	0.18	*Pgk1*	0.168	*Ywhaz*	2.34
4	*Hprt1*	0.29	*Rpl13a*	0.25	*Gapdh*	0.22	*Ppia*	0.218	*Hprt1*	3.31
5	*Gapdh*	0.32	*Ywhaz*	0.29	*Hprt1*	0.225	*Gapdh*	0.252	*Gapdh*	4.16
6	*Rpl13a*	0.41	*Hprt1*	0.3	*Rpl13a*	0.373	*Rpl13a*	0.305	*Rpl13a*	5.42

**Table A6:** Reference gene stability values within the ventral hippocampus of rats in the latent and chronic phases of the lithium–pilocarpine temporal lobe epilepsy model obtained by four algorithms with the RefFinder online tool.

Ventral Hippocampus										
	Delta CT		BestKeeper		NormFinder		GeNorm		Comprehensive Ranking	
Rank	Gene	Average of STDV	Gene	Std Dev	Gene	Stability Value	Gene	Stability Value	Gene	Geomean of Ranking Values
1	*Pgk1*	0.41	*Ppia*	0.32	*Pgk1*	0.089	*Hprt1 | Ywhaz*	0.203	*Pgk1*	1.73
2	*Ywhaz*	0.46	*Rpl13*	0.33	*Gapdh*	0.297			*Ywhaz*	2.51
3	*Hprt1*	0.48	*Gapdh*	0.34	*Ppia*	0.303	*Pgk1*	0.302	*Ppia*	2.94
4	*Gapdh*	0.48	*Pgk1*	0.34	*Ywhaz*	0.333	*Gapdh*	0.356	*Hprt1*	3.08
5	*Ppia*	0.49	*Ywhaz*	0.49	*Hprt1*	0.352	*Ppia*	0.399	*Gapdh*	3.36
6	*Rpl13a*	0.71	*Hprt1*	0.5	*Rpl13*	0.66	*Rpl13a*	0.504	*Rpl13a*	4.56
